# Supplementary material for: Syntenic Cell Wall QTLs as Versatile Breeding Tools: Intraspecific Allelic Variability and Predictability of Biomass Quality Loci in Target Plant Species
Source: Plants (Basel). 2023 Feb 9;12(4):779. doi: 10.3390/plants12040779 (PMC9961111; doi:10.3390/plants12040779)
Supplement: Supplementary file 1 [file plants-12-00779-s001.zip › plants-2201572-supplementary.pdf]

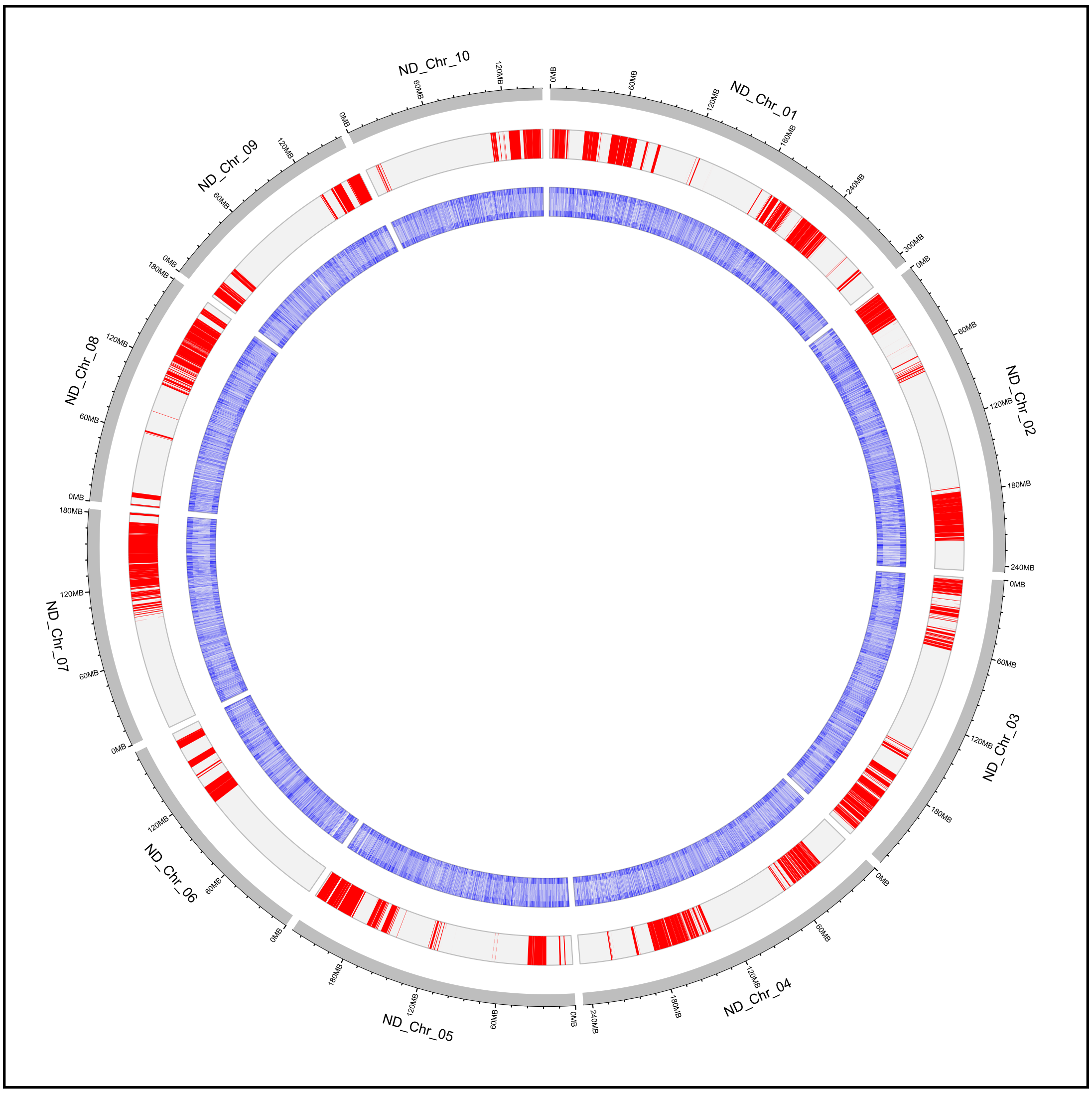

Supplementary Figure S1

Relative distribution of maize SQTLs (red regions) and maize transposons (as annotated by MaizeGDB onto the B73 genome v4.0) along the chromosomes of the maize B73 genome (v4.0).

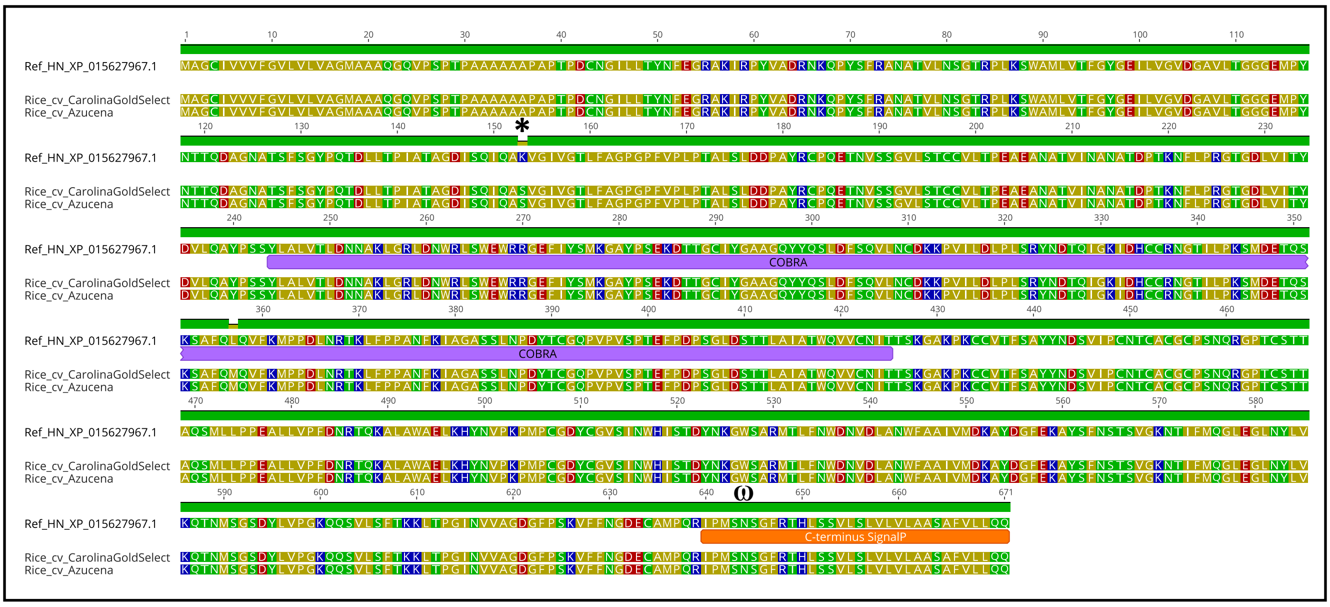

Supplementary Figure S2

Amino acid changes and their effects on the protein structure of *OsBCL1* between the reference rice cv. “Nipponbare” and the two cultivars “Azucena” and “CarolinaGoldSelect”. Coloring of amino acids reflects amino acid polarity, and protein domains and signal peptides are annotated. Amino acid changes indicated with * indicate a change in polarity, while sites annotated with **ω** indicate the predicted GPI-anchoring-related omega sites.

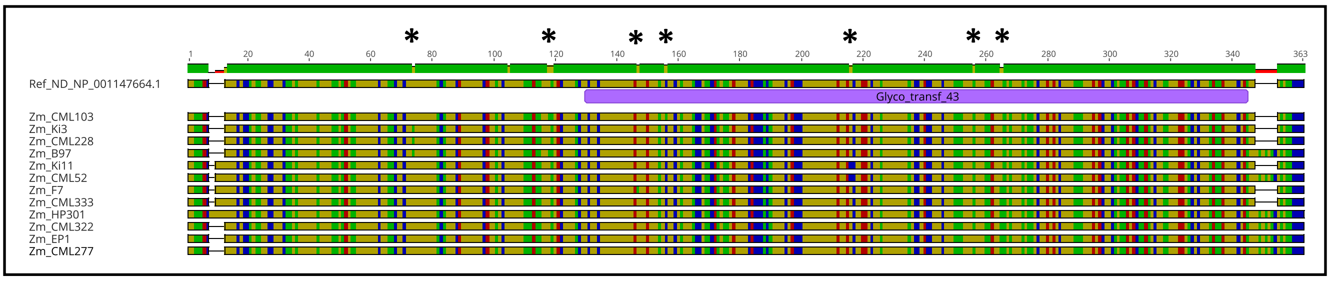

Supplementary Figure S3

Amino acid substitutions and INDELs of *ZmIRX9* between the reference maize B73 genome and 12 maize accessions. Coloring of amino acids reflects amino acid polarity, and the GT43 protein domain is annotated. Amino acid changes indicated with * indicate a change in polarity/charge in some of the accessions compared to the reference genome.

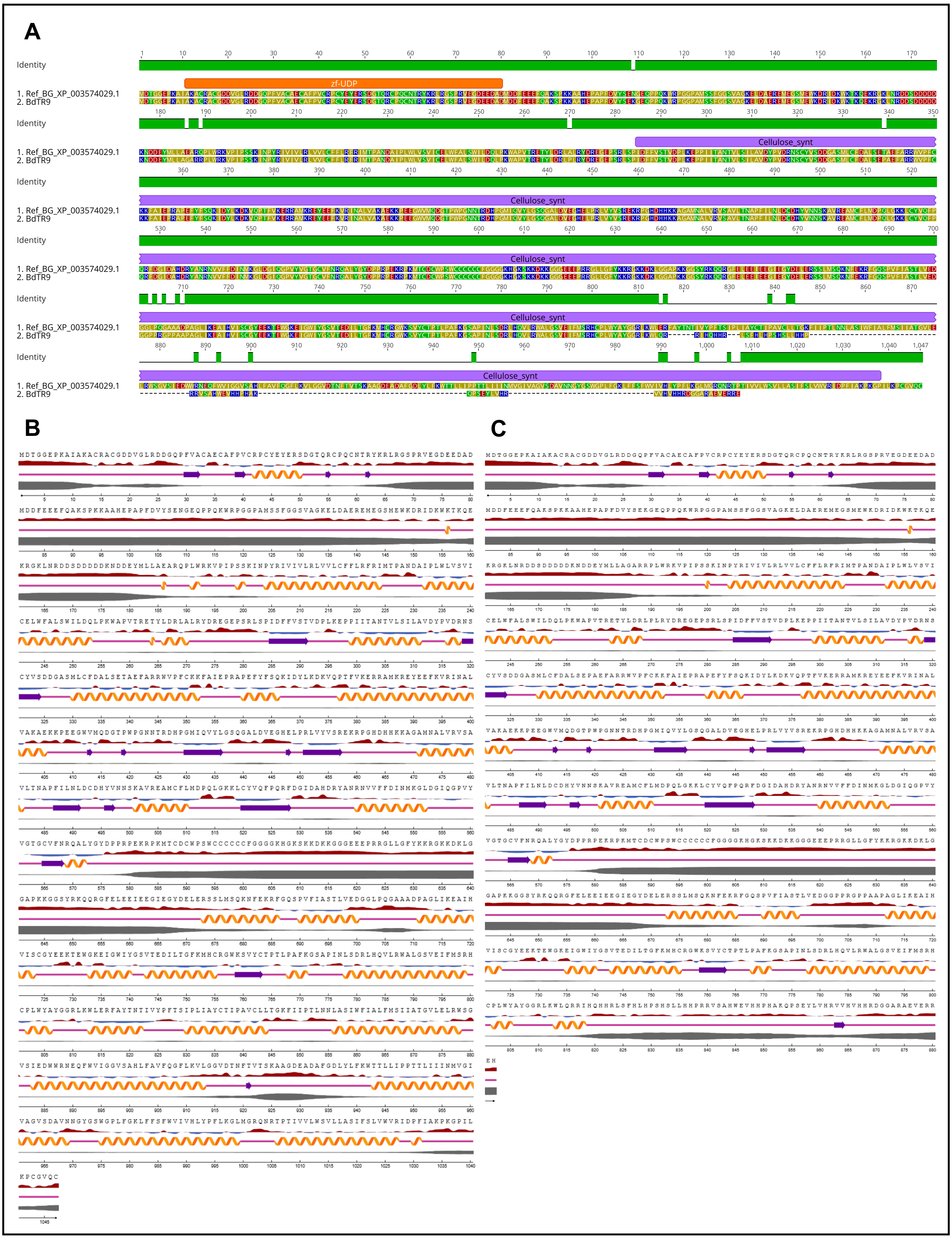

Supplementary Figure S4

Amino acid substitutions and INDELs of *BdCESA7* between the reference Brachypodium genome and the BdTR9 assembly. **A)** Multiple protein sequence alignment displaying substitutions and INDELs. Coloring of amino acids reflects amino acid polarity, and the Cellulose synthase protein domain is annotated. **B)** Predicted 2D protein structure of the reference BdCESA7 protein. **C)** Predicted 2D protein structure of the BdCESA7 protein from line BdTR9.

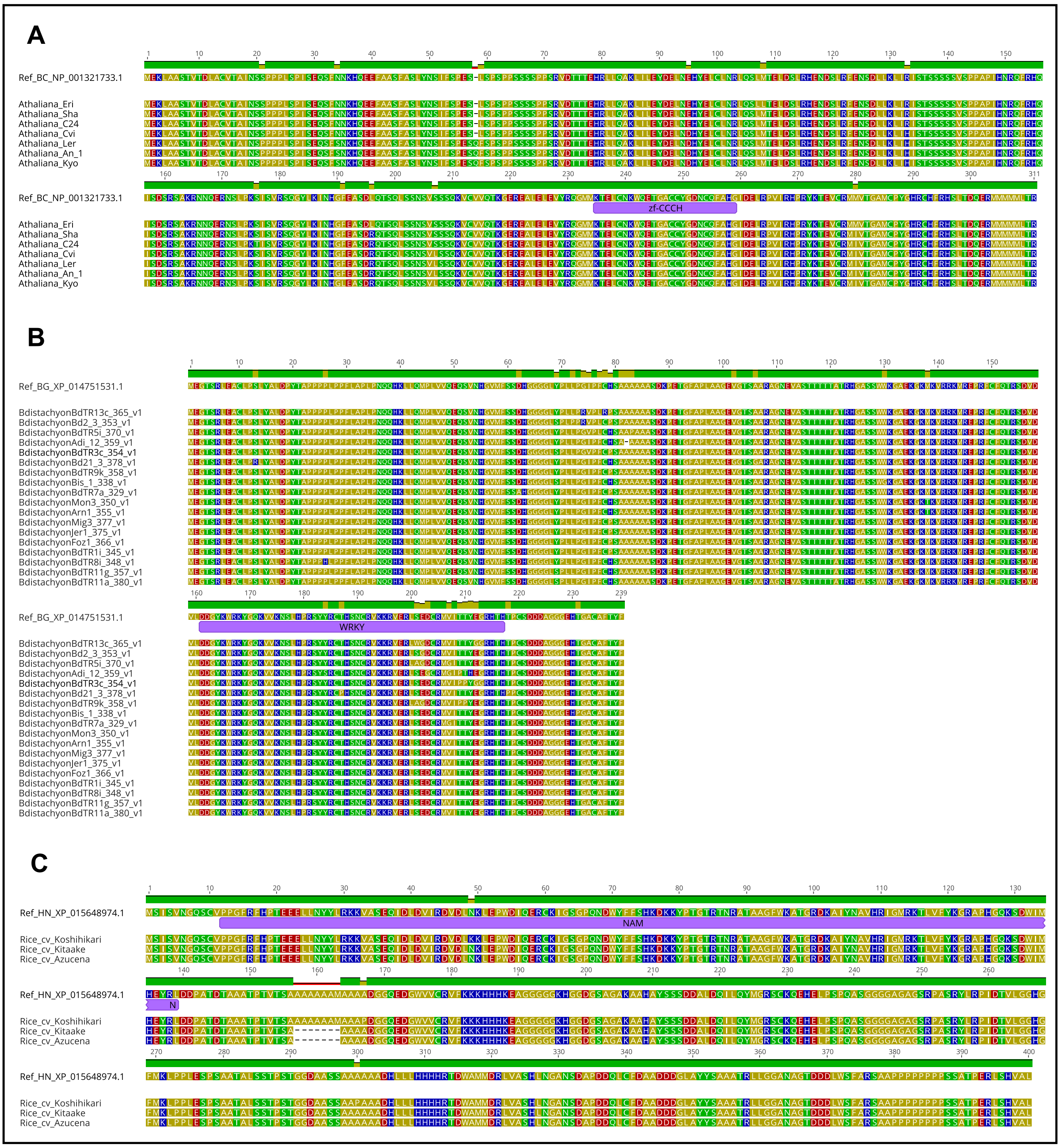

Supplementary Figure S5

Amino acid substitutions and INDELs of *AtC3H14*, *BdWRKY12*, and *OsNAC43* between the reference genome assemblies and multiple target genome assemblies displaying polymorphisms for the proteins coded by these genes. Coloring of amino acids reflects amino acid polarity, and the functional protein domains are annotated. **A)** *AtC3H14*; **B)** BdWRKY12; **C)** *OsNAC43*.

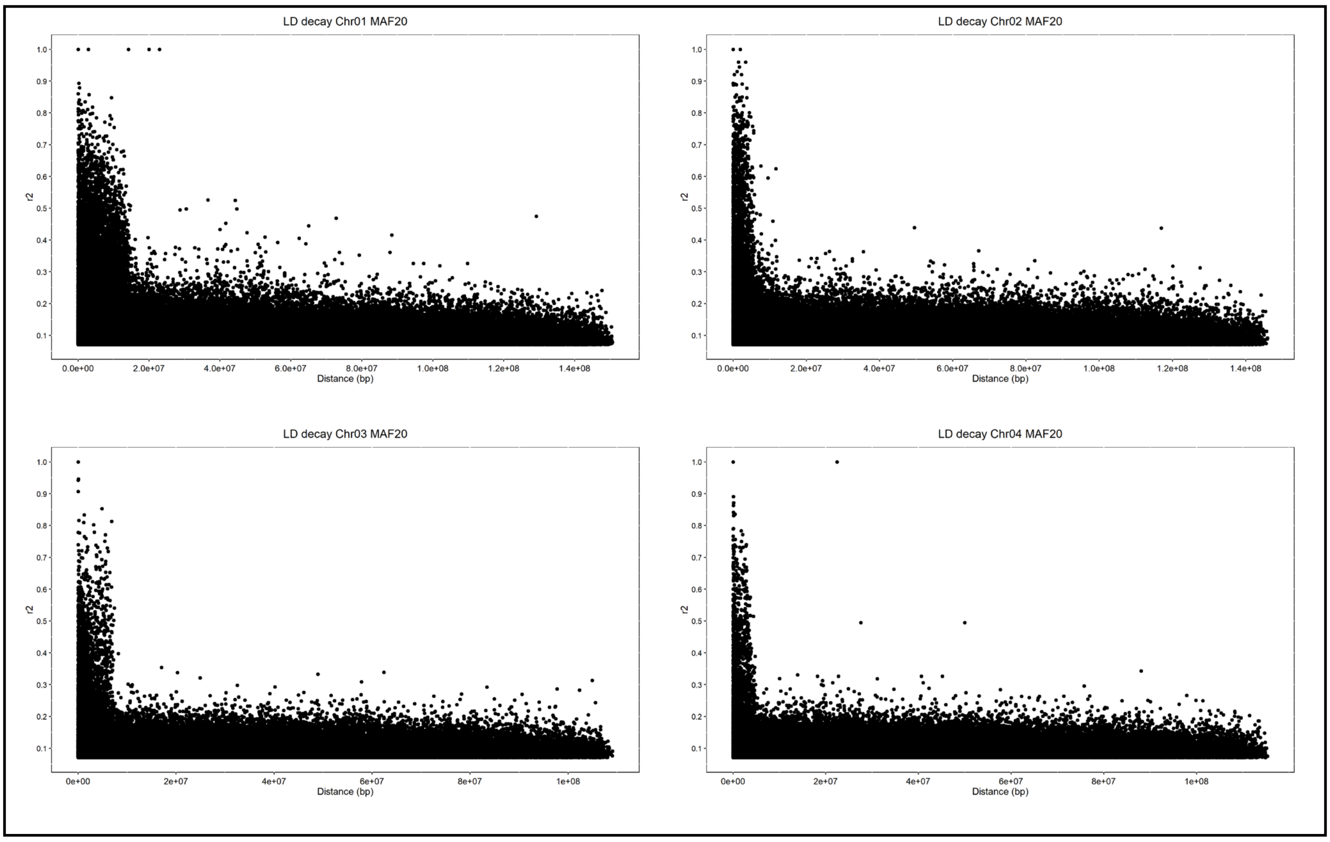

Supplementary Figure S6

Linkage disequilibrium decay plots of the first four Miscanthus sinensis chromosomes.

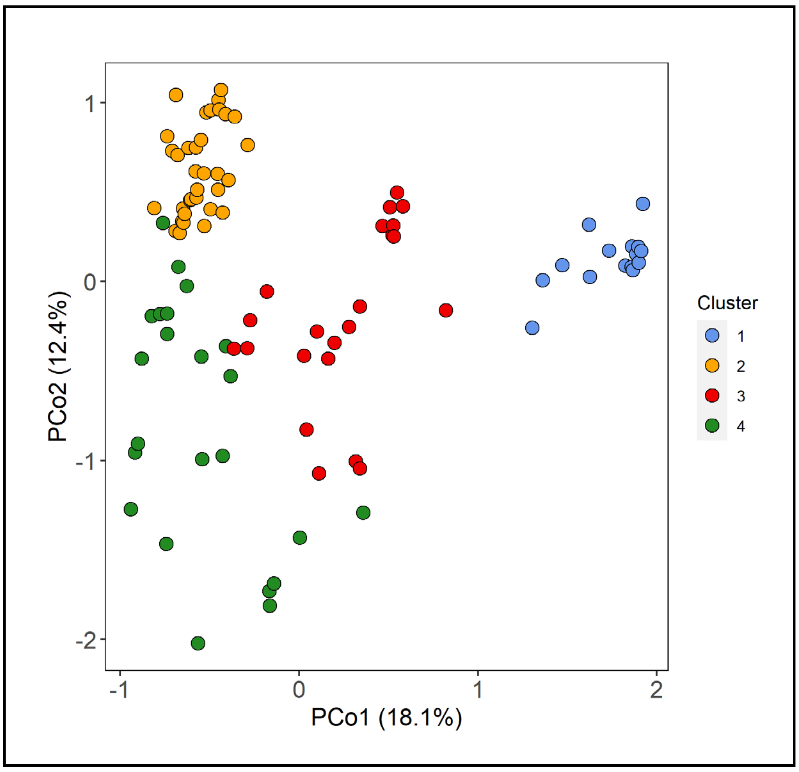

Supplementary Figure S7

PCoA of the kinship matrix of all the Miscanthus GWAS accessions, representing the population structure within the Miscanthus collection. Four main population structure groups emerge (different colors). The definition of the groups was based also on hierarchical clustering of the accessions based on SNP data.

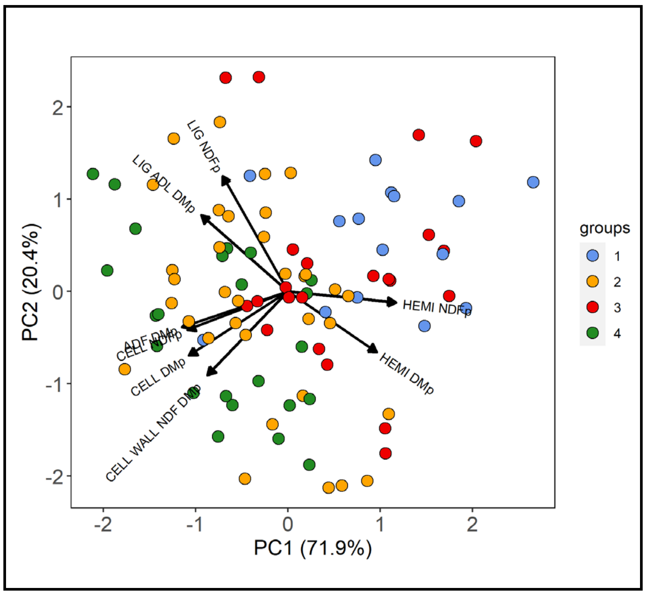

Supplementary Figure S8

PCA of Miscanthus GWAS accessions based on phenotypic data. Accession points are colored based on the four identified population structure groups (**Supplementary Figure 7**).

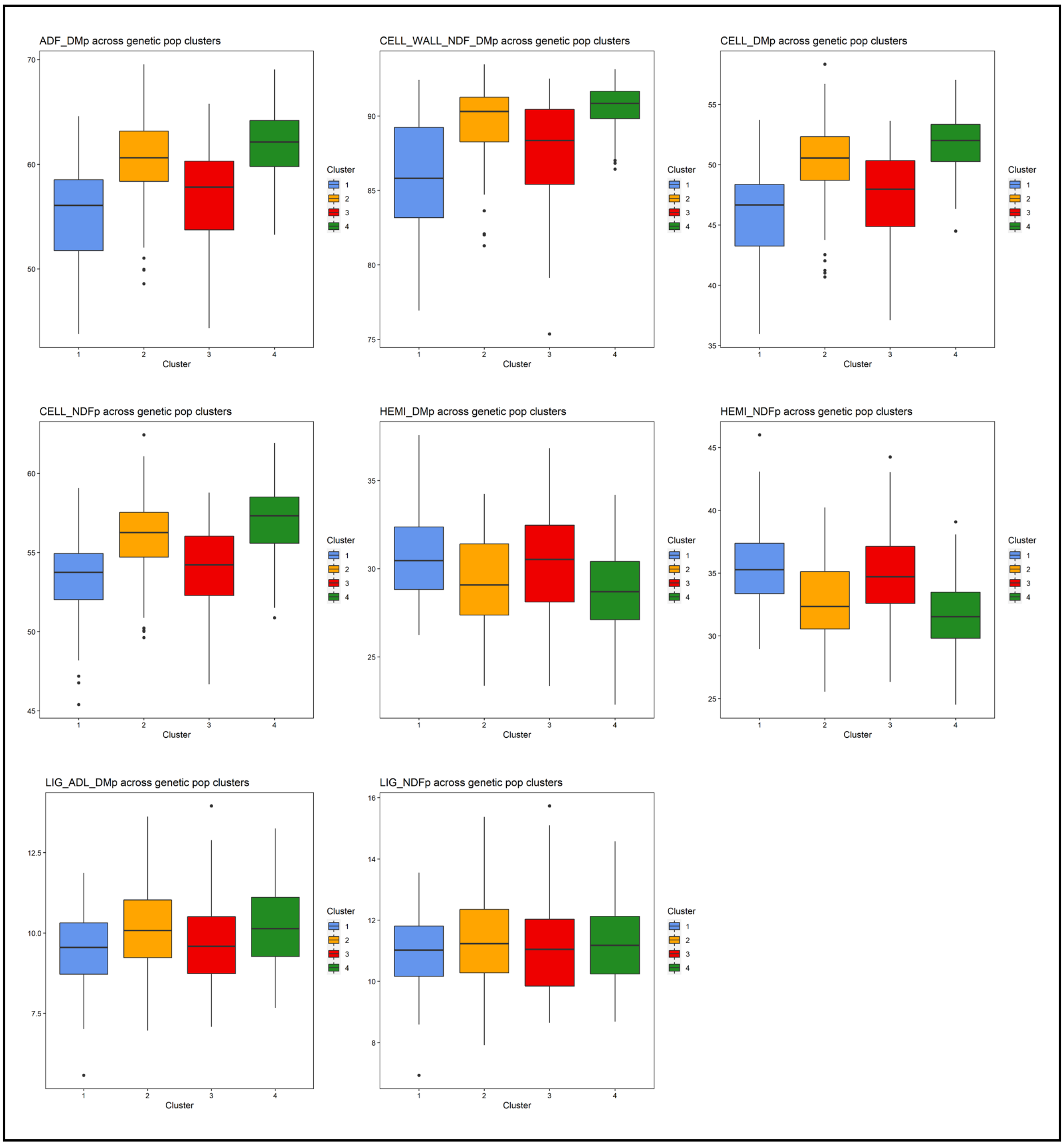

Supplementary Figure S9

Phenotypic variability per trait across the four population structure groups. Colors reflect the population structure groups defined as in **Supplementary Figure 7**.

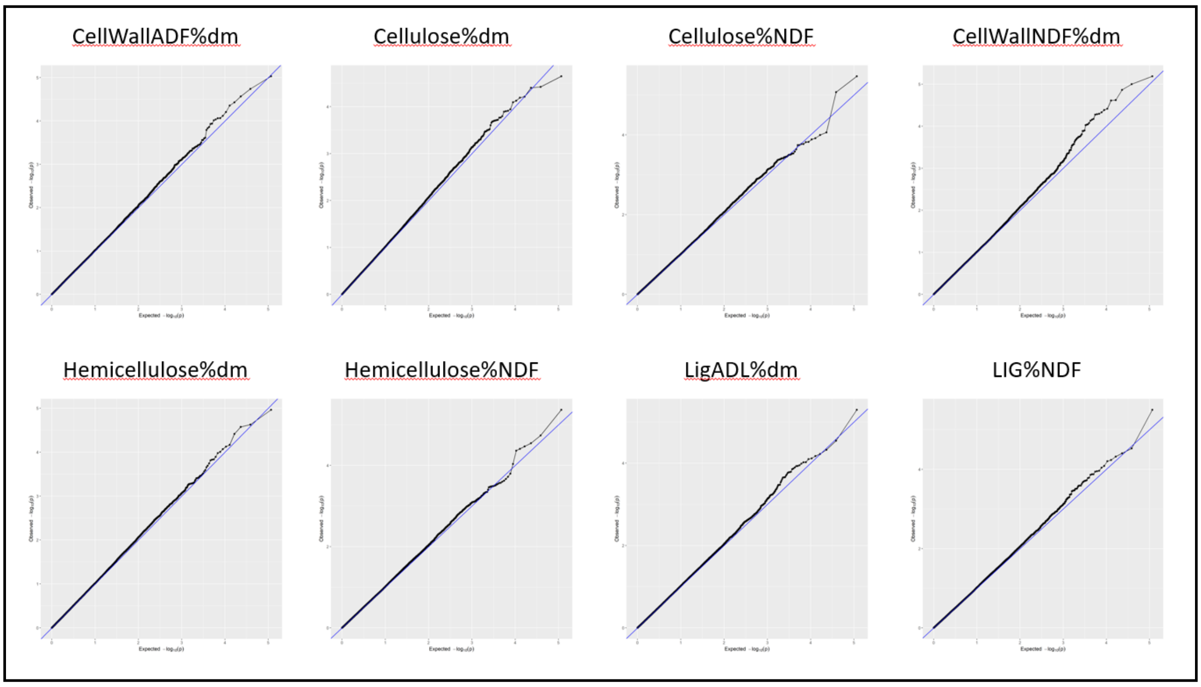

Supplementary Figure S10

QQ-plots of expected vs observed p-values of SNP associations after correction for population structure.
